# Genetic transformation of novel isolates of chicken *Lactobacillus *bearing probiotic features for expression of heterologous proteins: a tool to develop live oral vaccines

**DOI:** 10.1186/1472-6750-6-2

**Published:** 2006-01-05

**Authors:** Rodrigo M Mota, João Luiz S Moreira, Marcelo R Souza, M Fátima Horta, Santuza MR Teixeira, Elisabeth Neumann, Jacques R Nicoli, Álvaro C Nunes

**Affiliations:** 1 Departamento de Biologia Geral, Instituto de Ciências Biológicas, Universidade Federal de Minas Gerais, Av. Antônio Carlos 6627, 31.270-901, Belo Horizonte, MG, Brazil; 2 Departamento de Microbiologia, Instituto de Ciências Biológicas, Universidade Federal de Minas Gerais, Av. Antônio Carlos 6627, 31.270-901, Belo Horizonte, MG, Brazil; 3 Departamento de Bioquímica e Imunologia, Instituto de Ciências Biológicas, Universidade Federal de Minas Gerais, Av. Antônio Carlos 6627, 31.270-901, Belo Horizonte, MG, Brazil; 4 Centro Universitário Newton Paiva, Rua Goitacases 1762, 30.190-052, Belo Horizonte, MG, Brazil

## Abstract

**Background:**

The use of lactic acid bacteria as vehicles to delivery antigens to immunize animals is a promising issue. When genetically modified, these bacteria can induce a specific local and systemic immune response against selected pathogens. Gastric acid and bile salts tolerance, production of antagonistic substances against pathogenic microorganisms, and adhesive ability to gut epithelium are other important characteristics that make these bacteria useful for oral immunization.

**Results:**

Bacteria isolated on de Man, Rogosa and Sharpe medium (MRS) from different gastrointestinal portions of broiler chicks were evaluated for their resistance to artificial gastric acid and bile salts, production of hydrogen peroxide, and cell surface hydrophobicity. Thirty-eight isolates were first typed at species level by PCR amplification of 16S-23S rRNA intergenic spacers using universal primers that anneal within 16S and 23S genes, followed by restriction digestion analyses of PCR amplicons (PCR-ARDRA). An expression cassette was assembled onto the pCR2.1-Topo vector by cloning the promoter, leader peptide, cell wall anchor and terminator sequences derived from the laminin binding S-layer protein gene of *L. crispatus *strain F5.7 (*lbs *gene). A sequence encoding the green fluorescent protein (GFP) was inserted as reporter gene, and an erythromycin resistance gene was added as selective marker. All constructs were able to express GFP in the cloning host *E. coli *XL1-Blue and different *Lactobacillus *strains as verified by FACS and laser scanning confocal microscopy.

**Conclusion:**

*Lactobacillus *isolated from gastrointestinal tract of broiler chickens and selected for probiotic characteristics can be genetically modified by introducing an expression cassette into the *lbs *locus. The transformed bacteria expressed on its cell wall surface different fluorescent proteins used as reporters of promoter function. It is possible then that similar bacterial model expressing pathogen antigens can be used as live oral vaccines to immunize broilers against infectious diseases.

## Background

Probiotics are food or preparations containing live microorganisms, traditionally regarded as safe for human and animal use. When ingested in sufficient numbers, probiotics are believed to play an important role in the control of host intestinal microbiota and modulation of host immune responses [7]. Both local and systemic immune responses can be modulated by probiotics, with production of a set of cytokines such as IFN-γ, TNF-α, IL-6 and IL-12, and nitric oxide (NO) [8,26]. The increasing regulations and bans on the use of anticoccidial drugs in commercial poultry production, urges the need for novel approaches and alternative strategies to control avian eimeriosis. Probiotics are an alternative to antimicrobial drugs commonly employed as growth-promoter to broilers, avoiding drug residues to accumulate in animal carcasses and in the meat [11]. In ourdays, most of the probiotic preparations studied or commercialised contains lactic acid bacteria (LAB).

Criteria for screening of *Lactobacillus *strains for use as probiotics includes functional characteristics such as the ability to resist environmental conditions found in the digestive tract (low gastric pH and bile salts), to antagonize or competitively exclude pathogens by secretion of antimicrobial substances or to compete for nutrients and adhesion sites. LAB can produce different antimicrobial components such as organic acids, hydrogen peroxide, carbon peroxide, diacetyl, low molecular weight antimicrobial substances, bacteriocins, and adhesion inhibitor [24]. Production of hydrogen peroxide by *Lactobacillus *has been considered an important ecological factor that allows them to dominate some ecosystems like human vagina [20]. The adhesiveness of LAB may involve passive forces, electrostatic interactions, hydrophobic steric forces, lipoteicoic acids and lectins [24]. The hydrophobic nature of the outermost surface of microorganisms facilitates the adhesion of bacteria to host epithelium, conferring an advantage for competition and colonization in the gastrointestinal tract [28].

Besides their well known nutritional benefits and almost null pathogenicity, showed as millenary food supplements, the use of LAB as bacterial systems to express heterologous proteins or as vehicles to carry immunizing antigens after genetic modification is becoming a promising issue [18,22]. However, the successful expression of heterologous proteins in LAB depends on the promoter compatibility between the species or strains used as vector or hosts [13]. As pointed out by Pouwels et al. [18], the control of transcription and translation may differ greatly between two *Lactobacillus *species, implying that the knowledge generated for one organism may not simply be extrapolated to another. Genes that are efficiently expressed in one *Lactobacillus *species are not necessarily expressed in other species, or are expressed with different efficiency and/or with a different regulatory mechanism. Therefore, the correct typing of new isolates with probiotic properties is crucial for the development of a successful oral vaccine.

*L. crispatus *strains possess different S-proteins capable to bind proteins of intestinal extracellular matrix such as collagens (CbsA protein) and laminin (LbsA protein). The S-promoter responsible for the high level of transcription of stable mRNAs coding the S-protein monomers, which are capable to crystallizing into regular arrays and cover the *Lactobacillus *cell wall [5], is a good candidate to direct mRNA synthesis of chimerical genes for expression of heterologous proteins at the S-layer. The C-terminal one-third region of these S-proteins is responsible for the attachment to the peptidoglycan layer and incorporation of that sequence to chimerical proteins halts them to bacterial surface [25].

The aim of this study was to develop a transforming vector to express heterologous proteins using novel strains of *Lactobacillus *isolated from different gastrointestinal regions of broiler chickens and previously selected for some probiotic properties.

## Results

Thirty-eight isolates of bacteria were obtained from different gastrointestinal portions of five broiler chickens and selected as Gram-positive non-sporing, catalase negative, and presenting rods with diverse sizes. Table 1 summarizes the characteristics these isolates. Species identification by 16S-23S rRNA ARDRA showed that 7 different species were recovered in the following order: 12 *L. reuteri *(31,6%), 11 *L. acidophilus *(29,0%), 4 *L. johnsonii *(10,5%), 4 *L. salivarius *(10,5%), 4 *L. vaginalis *(10,5%), 2 *L. crispatus *(5,3%), and 1 *Lactobacillus *spp (2,6%). *L. reuteri *and *L. acidophilus *were more frequently isolated from gizzard and ceca, respectively. The highest biodiversity in term of number of different *Lactobacillus *species was found in intestines.

**Figure 1:**
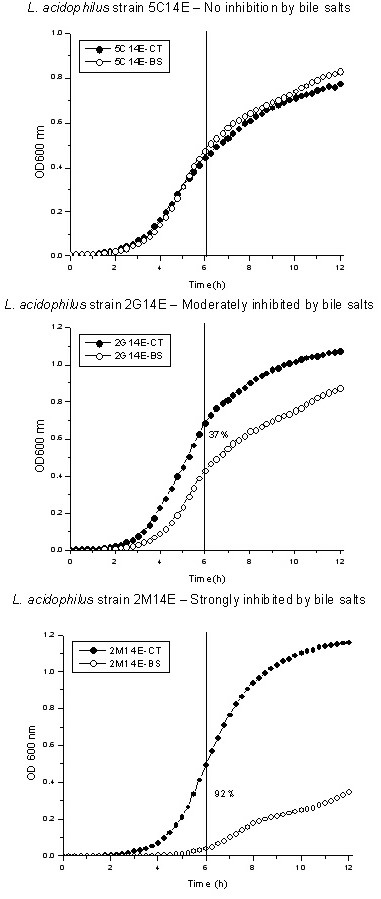
**Growth inhibition by bile salts of three different strains of *L. acidophilus***. *L. acidophilus *strains 5C14E (A), 2G14E (B) and 2M14E (C) were grown to an OD_600 nm _of 0.6 (logarithmic phase) and 1 % inoculated in MRS broth containing or not 0.3 % oxgall in a microtiter plate. Readings at OD_600 nm _were taken at 15 min intervals during 12 h of incubation at 37°C in a Microplate Spectrophotometer System SpectraMax 340. Percentage of growth inhibition was calculated after 6 h as (1 – A_BS_/A_CT_) × 100.

The strains were screened for the following probiotic criteria: *in vitro *gastric juice and bile salts resistance, surface hydrophobicity, and production of H_2_O_2_. All strains were highly resistant to acidic pH, showing low (about 5%) or no decrease of viable cell numbers 3 h after incubation at pH 2.5 (data not shown). On the other hand, bile salts tolerance varied markedly between strains (Table 1), even for same species (Fig. 1). Some isolates showed no or few growth inhibition in MRS broth supplemented with 0.3% oxgall, while others were moderately or highly susceptible to bile salts. The MATS (Microbial Adhesion To Solvents) method was used to evaluate the hydrophobic/hydrophilic cell surface properties of *Lactobacillus *isolates and to compare with those of other probiotic bacteria [16]. The bacterial adhesion to xylene at a high ionic strength of 0.1 M (pH 6.2) reflects cell surface hydrophobicity or hydrophilicity because electrostatic interactions are absent. Our results indicated that most of *Lactobacillus *isolates (79%) have hydrophobic surfaces (Table 1). Interestingly, all *Enterococcus *strains co-isolated in this study were highly hydrophilic (data not shown). The antagonistic capacity was evaluated by the production of H_2_O_2 _in TMB-Plus agar medium under anaerobic condition followed by air exposure. The results showed that most of *Lactobacillus *strains (75%) produced H_2_O_2 _(Table 1). The four isolates typed as *L. salivarius *(1D14C, 5G14C, 4C14C and 5C14C) produced more H_2_O_2 _than other species while seven *L. acidophilus*, one *L. johnsonii *(3G14L) and *L. vaginalis *(4G14E) were unable to produce H_2_O_2_.

**Figure 2:**
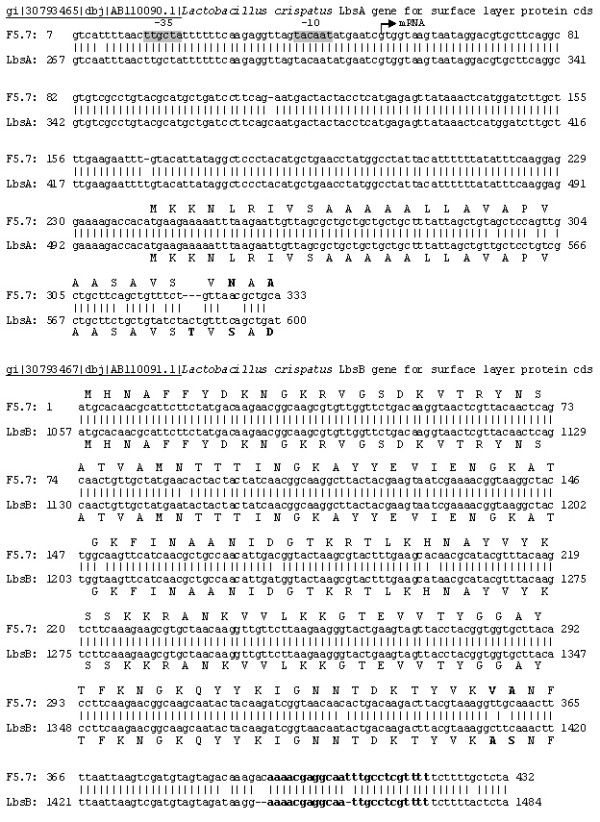
**Alignments of *Lactobacillus crispatus *Lbs gene sequences and PCR amplified sequences of *L. crispatus *strain F5.7 used to assemble the expression cassette of plasmid pLBS-GFP-Em^R^**. Upper panel shows the promoter plus leader peptide sequences aligned to LbsA gene and lower panel shows the C-terminal anchor plus terminator sequences aligned to LbsB gene.

**Figure 3:**
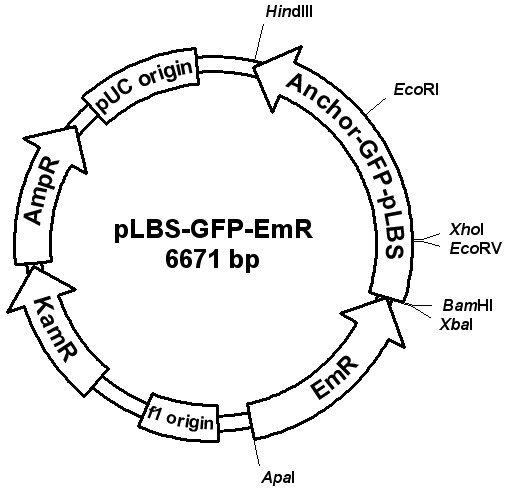
**Plasmid pLBS-GFP-Em^R ^constructed to transform chicken *Lactobacillus***. The promoter, leader peptide, C-terminal anchoring and terminator sequences of *lbs *gene of *L. crispatus *strain F5.7 were PCR amplified, cloned and assembled by overlapping restriction fragments onto pCR2.1-TOPO vector. The genes *erm*AM and *gfp *mut2 were also PCR amplified from other plasmids and used as selective marker and reporter gene, respectively.

In order to develop an expression vector for genetic manipulation of these new isolates of *Lactobacillus*, we PCR amplified the nucleotide sequences of the promoter, the leader peptide, the cell wall anchor and the terminator of *lbs *gene using genomic DNA of *L. crispatus *strain F5.7 (primers depicted in Table 2). As shown in Figure 2, a 344 pb fragment encompasses the regions containing the promoter, 5' untranslated portion and the leader peptide of the *lbs *gene. Figure 2 upper panel shows the alignment of this PCR product cloned into the pCR2.1-Topo and the sequence of the *lbs*A gene of *L. crispatus *(accession number AB110090). Figure 2 lower panel shows the alignment of the 600 bp PCR product encoding part of the coding region of *lbs*B gene that corresponds to the putative cell wall anchor and the terminator (accession number AB110091). These two fragments of the *lbs *gene were assembled into an expression cassette to produce recombinant proteins in fusion with the C-terminus of Lbs protein, which should allow the heterologous protein to be attached to the bacteria cell wall. The expression cassette was constructed into pCR2.1-Topo backbone opposite to the lac promoter (Fig. 3). This plasmid was chosen because it harbours replication origin for *E. coli *(pUC origin), which is not functional in Gram-positive bacteria such as *Lactobacillus *spp. Thus, we expected to integrate the expression cassette into the chromosome by homologous recombination within the S-layer protein locus, present in almost all species isolated from chicken [2]. The plasmid integration has probably occurred since we have not detected on gel electrophoresis of DNA extracted from transformed, erythromycin resistant and GFP-positive *Lactobacillus*, any DNA band that might correspond to plasmid DNA (-data not shown).

**Figure 4:**
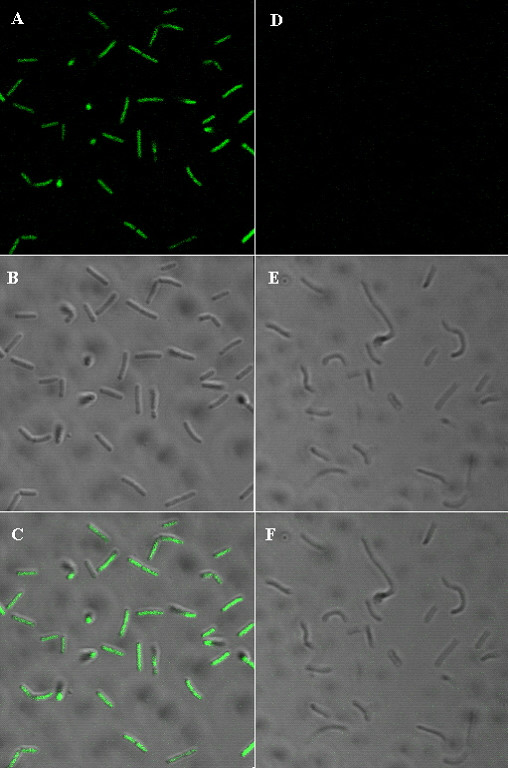
**Confocal scanning laser micrographs of GFP fluorescence of transformed *Lactobacillus delbrueckii***. (A – C) Genetically transformed lactobacillus with plasmid pLBS-GFP-Em^R^, and (D – F) control cells. Bacteria were grown overnight, washed, killed with sodium azide, and photographed under a laser scanning microscope (LSM) at scale of 54,8 × 54,8 μm. (C) Represents merged images of (A,B), and (F) represents merged images of (D,E).

The expression of *gfp *mut2 gene in *E. coli *XL1-Blue transformed with the pLBS-GFP-Em^R ^plasmid results in green fluorescent bacteria, as measured by FACS at 488 nm excitation wavelength (data not shown). The transformation of chicken *Lactobacillus *isolates F5.7, 1M14C, 1M14E and 3M14L, and the human strain of *L. delbrueckii *UFV H2b20, with the plasmid pLBS-GFP-Em^R ^was also successful as evaluated by PCR amplification of the *gfp *gene from the transformants (data not shown). However, the analysis by FACS was unsuccessful due to a strong green auto fluorescence displayed by all non-transformed control *Lactobacillus *when excited at 488 nm. To overcome this problem, we performed confocal microscopy analyses that could discriminate the fluorescence from the bacteria expressing GFP and the auto fluorescence using different excitation wavelengths. As shown in Figure 4, by exciting at 458 nm we were able to detect the control bacteria as non-fluorescent while the most of cells transformed with the pLBS-GFP-Em^R ^plasmid showed a strong green fluorescence.

## Discussion

In order to exert health-promoting effects, probiotic bacteria need to resist and survive the inhospitable conditions of chicken gastrointestinal tract. The fact that a high percentage of *Lactobacillus *cells adhered to xylene, an apolar solvent, demonstrated hydrophobic cell surface of these strains. However, some *Lactobacillus *isolates and all *Enterococcus *co-isolated strains showed more hydrophilic cell surface properties. Many previous studies on the physicochemistry of microbial cell surfaces have shown that the presence of (glyco-) proteinaceous material on the cell surface results in higher hydrophobicity, whereas hydrophilic surfaces are generally associated with the presence of polysaccharides [17]. The high hydrophobicity of most strains isolated in this prospecting study could facilitate bacteria-host cells interactions and improve antigen delivering to the immune cells present in the gastrointestinal associated lymphoid tissue (GALT).

The viability of probiotics is not an essential requirement for some antigen delivery since killed bacteria also stimulate the host immune system when ingested in sufficient numbers [16]. However, various biotherapeutic mechanisms are dependent on metabolic activity such as the direct competition for nutrients or secretion of antimicrobial substances. For the efficiency of an oral vaccine, cell viability is also probably fundamental. However, bacteria entering the gastrointestinal tract must be able to resist to certain local stresses such as the gastric pH and bile salts. In this context, the stress resistance showed by the *Lactobacillus *isolated in the present study would be important for these bacteria to exert a probiotic or oral live vaccine action. Since many pathogens, including *Eimeria *spp. sporozoites [10], are very sensitive to H_2_O_2_, its production by most of the *Lactobacillus *strains also could assure their success in the gut ecosystem and improve the vaccination process.

Non-pathogenic lactic acid bacteria (LAB) are attractive as live carriers to deliver protective antigens to the mucosal immune system. *Lactococcus lactis *is the LAB system for antigen delivery better established and used actually. Several molecular tools were constructed to allow antigen expression in three cellular locations, intracellular, secreted or anchored to the cell wall [3], combined to the adjuvant expression of IL-12 [4]. Such studies with a novel mucosal vaccine based on live Lactococci expressing E7 Antigen anchored to the cell surface and a secreted form of IL-12 showed that this prophylactic immunization can provide long-lasting immunity in mice against human Papillomavirus type 16-induced tumors and, also the therapeutic immunization with *L. lactis *recombinant strains induced regression of palpable tumors in treated mice a week after tumoral cells injection [4]. However, *Lactococcus lactis *is not a probiotic bacterium, being unable to adhere to the intestine cells and competitively exclude pathogens. Both persisting (*Lactobacillus plantarum *NCIMB8826/pMEC127) and non-persisting (*Lactococcus lactis *MG1363/pMEC46) strains of lactic acid bacteria have been evaluated by Grangette et al. [9] and seem to elicit a very strong specific and protective humoral response by the systemic and nasal routes of administration. The same authors have also shown that persistence and viability of the strain impacts on its immunogenicity and on the level of protection it may induce, indicating that *Lactobacillus *is more efficient than *Lactococcus *as a vaccine delivery vehicle [9]. Recently, the development of an efficacious vaccine against infection with *Helicobacter felis*, delivered by recombinant *Lactobacillus *strains producing *H. pylori *urease B (UreB) subunit, elicited UreB-specific antibodies and results in a decreased *H. felis *load in the stomachs of vaccinated mice. This was the first report of successful induction of partial protection against a pathogen with a mucosal prime-boost regimen in which recombinant *Lactobacillus *strains were used as antigen-delivery vehicles [6].

A new plasmid for genetic transformation of *Lactobacillus *species allowing expression of heterologous proteins attached to the cell wall components was successfully constructed into an *E. coli *plasmid vector backbone. This plasmid was able to transform *E. coli *XL1-Blue cloning host from non-fluorescent to green fluorescent cells, which could be measured by FACS analyses or easily visualized by standard fluorescence microscopy using fluorescein isothiocyanate (FITC) filter settings. Transformation of four different species of *Lactobacillus*, *L. crispatus*, *L. johnsonii*, *L. reuteri *and *L. delbrueckii*, from chicken or human origin, was also successful despite an unexpected strong green autofluorescence of non-transformed cells, reported here for the first time. Although the autofluorescence of native lactobacillus cells made FACS analyses or standard fluorescence microscopy not applicable, it could be abolished by exciting GFP at a wavelength lower than 488 nm using a laser confocal microscopy set at 458 nm. This could explain the constant use of the UV mutant of GFP by others researchers instead *gfp *mut2 or mut3 genes [8].

Usually, intrinsic problems related to the genetic modification could arise after the selection of novel isolates such as the presence of restriction/modification enzymes which could interfere with the establishment of new exogenous plasmids. Also, the presence of cryptic plasmids may result in incompatibility with the exogenous vector, as well as genetic exchange between plasmid from different bacteria present in the gastrointestinal tract. Our strategy to avoid these problems was to include in the characterization of the probiotic potential of the novel isolates, electrophoretic analyses for the presence of extrachromosomal DNA elements and to exclude those strains bearing endogenous plasmids. The persistence of the introduced plasmid was monitored through the detection of the new genetic traits such as the antibiotic resistance and *gfp *gene expression as well as by PCR analyses of the GFP sequence. Also, GFP expression was detected after growing for several generations in the absence of drug selection.

## Conclusion

Thirty-eight *Lactobacillus *strains were isolated from gastrointestinal tract of free-range chicks. Bacteria selected for some probiotic features (gastric juice and bile salts tolerance, high surface hydrophobicity and production of hydrogen peroxide) expressed the reporter protein GFP after transformation using a plasmid vector carrying an expression cassette assembled with *lbs *gene sequences. These results are currently being applied in our laboratories to develop an oral live vaccine to protect broiler chicks against eimeriosis, a major poultry disease.

## Methods

### Chickens

Five 14-day-old broiler chickens were raised under natural conditions and received commercial diet for pullets and water ad libitum, without any kind of medication (no vaccination, antibiotics, hormones or coccidiostatics).

### Bacteria isolation

Chickens were sacrificed by cervicaldislocation and gizzard, small intestine, large intestine, and ceca removed aseptically. In a laminar flow hood, samples were placed into sterile tubes containing 0.9% saline and homogenized with a glass rod. Decimal dilutions in saline were plated onto MRS agar and kept for 48 h at 37°C in anaerobiosis. Bacteria isolates, previously selected as described above (partially identified as pertaining to *Lactobacillus *genus), were cultured in de Man, Rogosa and Sharpe broth (MRS, Difco, Detroit, MI, USA) after inoculation with 1% of a fresh stationary culture and incubated in an anaerobic chamber (Forma Scientific Co., OH, USA) containing an atmosphere of 85% N_2_, 10% H_2 _and 5% CO_2_).

*L. crispatus *strain F5.7 was gently provided by Prof. Elinalva Maciel Paulo (Universidade Estadual de Feira de Santana, BA, Brazil), and the human strain UFV H2b20 of *L. delbrueckii *was gently provided by Dr. Célia Alencar de Moraes, Universidade Federal de Viçosa, MG, Brazil). *Escherichia coli *XL1-Blue was from Stratagene, CA, USA. *E. coli *was grown aerobically in Luria-Bertani (LB) medium. All the bacteria were stored at -80°C in respective broth with 30% glycerol.

### DNA extraction

Chromosomal DNA was isolated from overnight cultures of *Lactobacillus *sp in 10 ml MRS broth at 37°C. After washing the cells with 10 ml of deionised water, the pellet was resuspended in 1 ml of 5 M LiCl and incubated during 1 h under constant shaking. Cells were washed once more with 1 ml of deionised water and the pellet was suspended in 1 ml of protoplasting buffer (25 mM sucrose, 50 mM Tris HCl pH 8.0, 10 mM EDTA, 10 mg of lysozyme ml^-1^, 100 μg of RNaseA ml^-1^). After incubation during 1 h at 37°C and centrifugation at maximum speed in a microcentrifuge for 5 min, the pellet was resuspended in 500 μl of protoplasting buffer without sucrose and lysozyme, and 100 μl of 10% sodium dodecyl sulphate were added to allow cells lysis. The mixture was extracted once with phenol, with phenol-chloroform-isoamyl alcohol (25:24:1) and with chloroform-isoamyl alcohol (24:1). After isopropanol precipitation the DNA was dissolved in 100 μl of TE buffer.

### PCR-ARDRA of 16S-23S rRNA intergenic spacers

*Lactobacillus *isolates were identified to species-level by 16S-23S rRNA PCR-ARDRA according to Moreira *et al*. [14] using universal primers annealing into conserved regions of 16S and 23S rRNA genes [27]. PCR amplicons were digested by a set of 11 restriction enzymes and electrophoresed in a 1.4% agarose gel and visualized by UV transillumination after ethidium bromide staining. All restriction enzymes were purchased from Promega Corporation (Madison, WI, USA).

### Screening criteria for probiotic properties

Gastric juice tolerance: *Lactobacillus *stationary phase cultures were diluted 10 fold in simulated gastric juice (NaCl 2 g l^-1^, pepsin 3.2 g l^-1^, pH adjusted to 2.5 with concentrated HCl) and incubated at 37°C for 3 h. Bacteria viability was evaluated by plating 0.1 ml aliquots of serial dilutions from control (cells in saline) and gastric juice treated cells onto MRS agar. Colony forming units (cfu) were enumerated after incubation at 37°C during 24 h and the percentage of growth inhibition was calculated as (1 – log cfu_AGJ_/log cfu_CT_) × 100 [15], where cfu_AGJ _and cfu_CT _were the counts for simulated gastric juice and control, respectively.

Bile salts tolerance: Bile tolerance was evaluated according to Walker and Gilliland [29] adapted to microtiter plates. Briefly, *Lactobacillus *strains were fresh grown until an OD_600 nm _of 0.6 and 1% inoculated in MRS broth containing or not 0.3 % oxgall (Oxoid Co.) in a microtiter plate. Then, OD_600 nm _were determined at 15 min intervals during 12 h of incubation at 37°C in a Microplate Spectrophotometer System SpectraMax 340 (Molecular Devices, CA, USA). The percentage of growth inhibition was calculated as (1 – A_BS_/A_CT_) × 100 for 6 h of incubation.

Surface hydrophobicity: Microbial adhesion to solvents (MATS) was measured to evaluate the hydrophobicity of bacterial cell surface [12,17]. *Lactobacillus *stationary phase cultures were centrifuged, washed twice and adjusted to an OD_600 nm _of 0.6 with 0.1 M KNO_3_, pH 6.2 (A_0_). A volume of 0.2 ml of xylene was added to 1.2 ml of cell suspension and after a 10 min pre-incubation at room temperature, the two-phase system was mixed on a vortex for 2 min. The aqueous phase was removed after 30 min and its OD_600 nm _was measured (A_1_). The percentage of MATS was calculated as (1 – A_1_/A_0_) × 100. Percentage values smaller than 50% were considered as hydrophilic, and values higher than 50% as hydrophobic.

Hydrogen peroxide formation: H_2_O_2_-producing strains were identified by an agar medium assay optimised to detect H_2_O_2 _production according to Rabe & Hillier [19]. Briefly, 2 μl of *Lactobacillus *stationary phase cultures were spotted onto the surface of TMB-Plus agar supplemented with 3,3',5,5'-tetramethylbenzidine (TMB) and horseradish peroxidase, and incubated at 37°C for 18 h in an anaerobic chamber. Then, plates are exposed to air for 30 min, and a blue pigment appears if H_2_O_2 _was produced. The intensity of blue colour was assigned as +++ if was dark blue (almost black), ++ if was blue, + if was bluish generally with a blue border, and – if colour was absent.

### Cloning of the PCR-amplified products

The typical reaction mixture contained 10 pmol of each primer, 0.2 mM of each deoxyribonucleotide triphosphate, reaction buffer with 1.5 mM MgCl_2_, 5 U of *Taq *DNA polymerase (Phoneutria Biotecnologia & Serviços, Brazil), and 10 ng of template DNA. PCR products were cleaned-up using the Concert™ Rapid PCR Purification System and cloned in *E. coli *XL1-Blue into pCR2.1-TOPO vector using the Invitrogen TOPO TA cloning kit (Invitrogen Life Technologies, Carlsbad, USA). PCR was performed on cell lysates of ampicillin-resistant transformants using M13 specific primers to confirm the size of the inserts. Inserts were digested with restriction enzymes, excised from the gel and purified with the Concert™ Rapid Gel Extraction System according to the manufacturer's instructions (Invitrogen Co.).

### Plasmid DNA preparation and transformation

Plasmid DNA from *E. coli *was isolated using the commercial kit GFX™ Micro Plasmid Prep kit (Amersham Biosciences, Piscataway, NJ, USA) by alkaline lysis method. *E. coli *XL1-Blue was used as host strain for the construction of the plasmids, and chemically competent cells were transformed according to the manufacturer's procedure. *Lactobacillus *strains were electrotransformed with 1–2 μg of pLBS-GFP-Em^R ^plasmid according to the optimised procedure of Serror *et al*. [23].

### Plasmid constructions

Plasmids were constructed using standard molecular cloning and PCR fusion (overlap extension) techniques [21]. Primers were purchased from Invitrogen Life Technologies (São Paulo, Brazil) and Integrated DNA Technologies, Inc. (Coralville, IA, USA), and are listed in Table 1. Plasmid pLBS-GFP-Em^R ^(Fig. 3) was designed to contain the 'backbone' elements of the cloning vector pCR2.1-Topo: the replication determinants (pUC ori), and ampicillin and kanamycin resistance markers for *E. coli*. The *Lactobacillus *specific sequences were PCR amplified from genomic DNA of *L. crispatus *strain F5.7 to assemble the expression cassette containing the *lbs *promoter, leader peptide, anchor and terminator sequences. Homologous sequences to the anchor region of Lbs are found in other S-layer proteins from different *Lactobacillus *species, such as Slp of *L. acidophilus *(accession X89375, X89376), SlpH of *L. helveticus *(accession X91199, X92752), Lgs of *L. gallinarum *(accession AY597259, AY597266), Slp of *L. suntoryeus *(accession AY641395), Cbs of *L. crispatus *(accession AF001313, AF079365), with nucleotide identities higher than 80% to 90%. These nucleotide stretches can be enough to allow plasmid integration at *lbs *locus via homologous recombination, otherwise it will be unable to propagate in *Lactobacillus*. An erythromycin resistance marker (*erm*AM) was amplified from plasmid pRV566. The *Aequorea victoria *GFP was PCR amplified from commercial plasmid (Clontech, Palo Alto, CA, USA) and used as reporter gene to test the expression of heterologous proteins.

### Sequencing and sequence analysis

DNA sequencing was carried out at the Núcleo de Análise de Genoma e Expressão Gênica (NAGE), Instituto de Ciências Biológicas, UniversidadeFederal de Minas Gerais, Belo Horizonte, MG, Brazil, using a DYEnamic™ ET Dye Terminator Cycle Sequencing kit for MegaBACE™ DNA Analysis Systems (Amersham Biosciences, USA) in combination with a MegaBACE™ 1000 automated sequencing system. Both polynucleotide strands of the cloned DNA were sequenced, using M13 forward and reverse primers. The sequences obtained were compared to sequences held in GenBank DNA database using the BLAST algorithm [1].

### Confocal scanning laser microscopy

Confocal microscopy work was performed using a LSM 510 META inverted confocal scanning laser microscope (Carl Zeiss Ltd., Germany) carried out at the Centro de Microscopia (CEMEL), Instituto de Ciências Biológicas, Universidade Federal de Minas Gerais, Belo Horizonte, MG, Brazil. Randomly selected areas of each sample were imaged using a ×63 magnification objective with a numerical aperture of 1.4. Confocal illumination was provided by a Kr/Ar laser (458-nm laser excitation) fitted with a long-pass 520-nm emission filter (greenfluorescence signal).

## Authors' contributions

RMM, JLSM and MRS carried out all the experiments: dissection of chicks, bacteria isolation, DNA extraction, PCR amplification, gel electrophoresis, restriction digestion, and cloning. MFH and SMRT participated to the discussion of the results, and the manuscript draft. EN and JRN participated to the study design. ACN conceived and designed the study, and coordinated and participated to the manuscript draft. All authors read and approved the final manuscript.

## Figures and Tables

**Table 1:** *Lactobacillus *strains isolated from different portions of chicks gastrointestinal tract, species identification by PCR-ARDRA and some probiotic features

Isolate	Species identification^a^	Bile salt inhibition (%)^b^	Hydrophobicity (%)^c^	H_2_O_2 _production^d^
Gizzard				
1M14C	*L. reuteri*	14	89	nd^e^
1M14E	*L. johnsonii*	59	35	+
2M14C	*L. reuteri*	21	58	++
2M14L	*L. acidophilus*	58	52	+
2M14E	*L. acidophilus*	92	88	+
3M14C	*L. reuteri*	25	42	+
3M14L	*L. johnsonii*	24	61	+
4M14C	*L. reuteri*	19	64	+
4M14L	*L. reuteri*	54	36	+
4M14E	*L. acidophilus*	96	5	-
5M14C	*L. reuteri*	18	78	+
5M14E	*L. vaginalis*	100	75	++
Small intestine				
1D14C	*L. salivarius*	13	60	+++
2D14C	*L. vaginalis*	71	67	++
2D14E	*L. acidophilus*	46	76	-
3D14C	*L. reuteri*	13	83	++
3D14L	*L. crispatus*	36	88	++
4D14C	*L. reuteri*	13	88	+
4D14L	*L. reuteri*	20	57	+
5D14E	*L. acidophilus*	0	83	-
Large intestine				
1G14E	*Lactobacillus*	27	78	-
2G14E	*L. acidophilus*	37	55	-
3G14C	*L. reuteri*	14	67	++
3G14L	*L. johnsonii*	22	45	-
4G14C	*L. vaginalis*	37	25	++
4G14L	*L. reuteri*	40	35	++
4G14E	*L. vaginalis*	100	72	-
5G14C	*L. salivarius*	43	66	+++
5G14L	*L. crispatus*	41	35	+
Ceca				
2C14E	*L. acidophilus*	72	55	-
2C14L	*L. acidophilus*	10	78	-
3C14C	*L. reuteri*	0	84	++
3C14L	*L. johnsonii*	24	83	+
3C14E	*L. acidophilus*	80	64	+
4C14C	*L. salivarius*	43	92	+++
4C14L	*L. acidophilus*	73	79	nd
5C14C	*L. salivarius*	41	83	+++
5C14E	*L. acidophilus*	0	64	+

^a^Isolates typed by 16S-23S rRNA PCR-ARDRA according to Moreira *et al*. (2005)

^b^Percentage of optical density reduction after 6 h growing in MRS containing 0.3% bile salts

^c^Percentage of microbial adhesion to xylene

^d^H_2_O_2 _production in TMB-Plus (+++, dark blue; ++, blue; +, light blue/blue border; -, none)

^e^Not determined

**Table 2:** Primers used to amplify the portions of S-layer protein gene of *Lactobacillus crispatus *(*lbs*), the green fluorescent protein gene (*gfp *mut2), and the erythromycin gene (*erm*AM) used as selective marker, respectively.

Primer sequence	Amplicon	Size (bp)
5'**GGATCC**CGGTCATTTTAACTTGCTA 3'		
5'**CTCGAGGATATC**TGCAGCGTTAACAGAAACAGCTG 3'	*lbs p*romoter plus leader peptide	344
5'**GAATTC**ATGCACAACGCATTCTTCTATG 3'		
5'**AAGCTT**CAGAAGATCCTATTAGAACTGTATGTTTAG 3'	*lbs *anchor plus terminator	600
5'**CTCGAG**GCTAGTAAAGGAGAAGAACTTTTCACT 3'		
5'**GAATTC**TGCTTTGTATAGTTCATCCATGCCATG 3'	*gfp *mut2	720
5'**GGGCCC**TCTAGCACAAAAAGAAAAACG 3'		
5'**GGATCCTCTAGA**GTCTAGGGACCTCTTTAGC 3'	*erm *AM	1200
